# FAK loss reduces BRAF^V600E^-induced ERK phosphorylation to promote intestinal stemness and cecal tumor formation

**DOI:** 10.21203/rs.3.rs-2531119/v2

**Published:** 2024-01-05

**Authors:** Chenxi Gao, Huaibin Ge, Shih-Fan Kuan, Chunhui Cai, Xinghua Lu, Farzad Esni, Robert Schoen, Jing Wang, Edward Chu, Jing Hu

**Affiliations:** University of Pittsburgh; University of Pittsburgh; University of Pittsburgh; University of Pittsburgh; University of Pittsburgh; University of Pittsburgh; University of Pittsburgh; UPMC Hillman Cancer Center/University of Pittsburgh; Albert Einstein Cancer Center; University of pittsburgh

**Keywords:** FAK, BRAF mutation, EGFR, ERK, carcinogenesis

## Abstract

*BRAF* ^V600E^ mutation is a driver mutation in the serrated pathway to colorectal cancers. BRAF^V600E^ drives tumorigenesis through constitutive downstream extracellular signal-regulated kinase (ERK) activation, but high-intensity ERK activation can also trigger tumor suppression. Whether and how oncogenic ERK signaling can be intrinsically adjusted to a “just-right” level optimal for tumorigenesis remains undetermined. In this study, we found that FAK (Focal adhesion kinase) expression was reduced in *BRAF*^V600E^-mutant adenomas/polyps in mice and patients. In *Vill-Cre*;*BRAF*^V600E/+^;*Fak*^fl/fl^ mice, *Fak* deletion maximized BRAF^V600E^’s oncogenic activity and increased cecal tumor incidence to 100%. Mechanistically, our results showed that Fak loss, without jeopardizing BRAF^V600E^-induced ERK pathway transcriptional output, reduced EGFR (epidermal growth factor receptor)-dependent ERK phosphorylation. Reduction in ERK phosphorylation increased the level of Lgr4, promoting intestinal stemness and cecal tumor formation. Our findings show that a “just-right” ERK signaling optimal for *BRAF*^V600E^-induced cecal tumor formation can be achieved via Fak loss-mediated downregulation of ERK phosphorylation.

## Introduction

Colorectal cancer (CRC) is a heterogeneous disease arising through several discrete evolutionary pathways. The best-known and most-studied pathway to CRC is the canonical pathway, in which cancer originates from conventional adenomatous polyps bearing *APC* (*adenomatous polyposis coli*) mutation ^1,2^. Recently a new “alternative” pathway through serrated adenoma—the serrated pathway —has been uncovered. Mice studies have established that the *BRAF*^V600E^ mutation is a driver mutation in the serrated pathway ^3–5^. In patients, *BRAF*^V600E^ mutation is found in 50–67% of serrated CRC ^6^ and 10–15% of all CRCs ^7^.

The “Goldilocks principle” applies to mutant *APC*-driven and mutant *BRAF*-driven intestinal tumorigenesis: a threshold of oncogenic signaling needs to be reached for dysplastic lesions to form, but optimum tumor development requires “just-right” levels of oncogenic signaling, with too much being as detrimental as too little. In the canonical pathway to CRC, the primary driving force is mutant APC-mediated activation of Wnt/b-catenin signaling ^8^, and the “just-right” level of Wnt/b-catenin signaling optimal for tumor formation is achieved mainly by the selection for specific APC mutant proteins based on their residual b-catenin-downregulating activity ^9–12^. The selection for *APC* mutations in the intestine is influenced by the underlying basal/physiological level of Wnt activity and stemcell number, and *APC* mutation spectra vary throughout the intestinal tract resulting in different *APC* mutation spectra in the proximal and distal CRCs ^10,11^. In addition to the different mutation spectra, the ‘optimal’ thresholds for proximal and distal cancers are also variable ^11^.

BRAF^V600E^ drives tumorigenesis through constitutive downstream ERK1/2 activation ^13^, but hyperactivation of ERK induced by oncogenic BRAF^V600E^ is not tolerated in the intestine: high ERK activation, induced by transgenic expression of oncogenic BRAF (BRAF^V600K^) or by activation of two BRAF alleles in *BRAF*^V600E/V600E^ mutant mice, engages tumor suppressive mechanisms, causing loss of stem cells and induction of differentiation and senescence ^14,15^. Lowering ERK activation by treatment with ERK or MEK (mitogen-activated protein kinase kinase) inhibitor counteracted BRAF^V600E^-induced organoid disintegration ^14,16^. It is therefore presumed that maintaining ERK activation within a narrow threshold range to avoid engaging tumor suppression is pivotal for mutant BRAF to exhibit the strongest transforming activity. However, despite being highly anticipated ^16^, the existence of *in vivo* intrinsic fine-tuning of mutant *BRAF*-induced ERK activation has never been experimentally examined. Given that over 60 mutations have now been identified in *BRAF* ^13,17^, theoretically, mutation selection could be a way to achieve optimal ERK activation. However, because the V600E mutation accounts for about 90% of BRAF mutation seen in human cancer ^18^, mutation selection is not the primary means to achieve the “just-right” levels of oncogenic ERK signaling. Normally, ERK activation is self-limiting by the rapid inactivation of upstream kinases and delayed induction of dual-specific MAKP phosphatases (MKPs/DUSPs) ^19^. Although feedback inhibitors of ERK signaling, including DUSPs are overexpressed in BRAF^V600E^-expressing cells, the ERK signaling pathway is refractory to upstream feedback inhibition ^20^. EGFR is a core receptor upstream of the MAPK kinase axis. *In vitro* cell culture studies show that all activating BRAF mutants are RAS-independent ^21^: neither RAS inhibition ^21^ nor EGFR inhibition ^22,23^ was able to inhibit mutant-BRAF-induced ERK phosphorylation in *BRAF*-mutant human CRC cell lines.

In this study, we addressed whether BRAF^V600E^-induced ERK activation is still tuneable during tumorigenesis *in vivo*. If yes, what are the factors involved in the regulation? Can BRAF^V600E^-induced ERK activation be fine-tuned to a “just-right” level optimal for tumor initiation? Our study identified FAK as a key regulator of BRAF^V600E^-induced ERK activation in mutant *BRAF*-induced serrated tumor formation/initiation and revealed that FAK loss allows BRAF^V600E^-induced ERK signaling to reach the permissive threshold “just-right” for cecal tumors to form.

## Results

### FAK expression is reduced in *BRAF*^V600E^-mutant serrated lesions in humans and mice.

**Figure 1:**
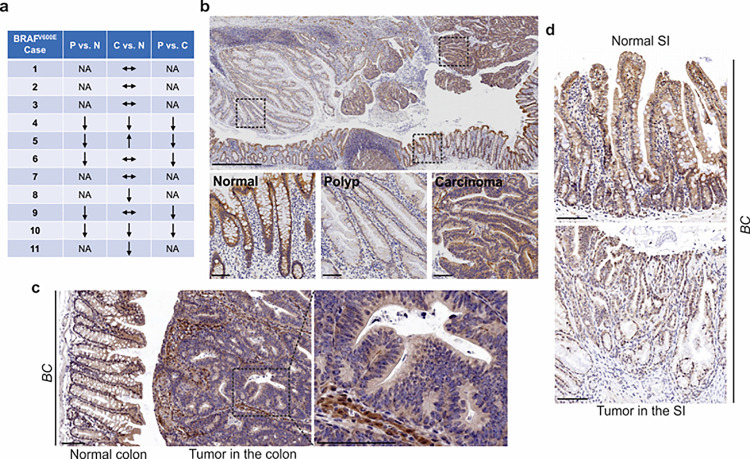
FAK downregulation in serrated tumors. **a** Summary of FAK IHC staining in 11 human *BRAF*^V600E^-mutant CRC samples. N represents normal colon; P represents polyp; C represents carcinoma; NA, not applicable; ↔ represents no change; ↑ represents an increase. ↓ represents a decrease. **b** Representative IHC staining of *BRAF*^V600E^-mutant patient SSA/P, serrated colorectal adenoma, and adjacent normal tissues. **c** IHC staining of Fak in small intestine tumors in a 12-month-old *BC* mouse. **d** Representative IHC staining of Fak in colon tumor in 12-month-old *BC* mice. Scale bars in **b**: 1 mm (upper panel) and 100 μm (lower panel). Scale bars in **c** and **d**: 100 μm.

FAK is a cytoplasmic non-receptor tyrosine kinase involved in many aspects and types of cancer ^24^. To determine the role of FAK in mutant *BRAF*-induced serrated CRC, we first evaluated FAK protein expressions in human *BRAF*^V600E^-mutated serrated tumors (11 cases). We examined tissue sections containing *BRAF*^V600E^-mutant CRCs, sessile serrated adenoma/polyps (SSA/P)s, and adjacent histologically normal colon from the same tissue block. Results of immunohistochemistry (IHC) staining showed that FAK protein levels were lower in SSA/Ps (5/5) than in normal intestines and CRCs (5/5) (Fig. 1a). FAK expression was more complex in CRCs. FAK levels in CRCs were either similar to (6/11) or lower (4/11) or higher (1/11) than that of the normal intestines (Fig. 1a, b). FAK was mainly localized in the cytoplasm (Fig.1b). In mice, compared to the neighboring normal mucosa or stroma in the tumor, Fak protein levels were substantially decreased in carcinomas in the colon (Fig. 1c) and adenomas/polyps in the small intestine (SI) (Fig. 1d) in *Vill-Cre*;*BRAF*^V600E/+^ (*BC*) mice. The downregulation of FAK in human and mouse polyps suggests that FAK loss may play a role in *BRAF*^V600E^-induced tumor formation/initiation.

### Fak deletion promotes *BRAF*^V600E^-induced cecal tumor formation.

Previous mice studies show that *Fak* deletion suppresses mammary tumorigenesis ^25,26^, mutant *Apc*-induced intestinal tumorigenesis ^27^, skin tumor formation ^28^, and hepatocarcinogenesis ^29^. To address the functional significance of FAK downregulation in *BRAF*^V600E^-induced serrated tumor formation/initiation, we generated the *Vill-Cre*;*BRAF*^V600E/+^;*Fak*^fl/fl^ (*FBC*) mice. The Cre-mediated recombination efficiency was confirmed by tdTomato-reporter expression in intestinal crypts in *Vill-Cre*;*Rosa26*^LSL-tdTomato/+^ mice (Supplementary Fig. 1a). Deletion of Fak in the intestinal epithelium was further confirmed by IHC staining of the intestine in *FBC* mice (Supplementary Fig. 1b).

**Figure 2:**
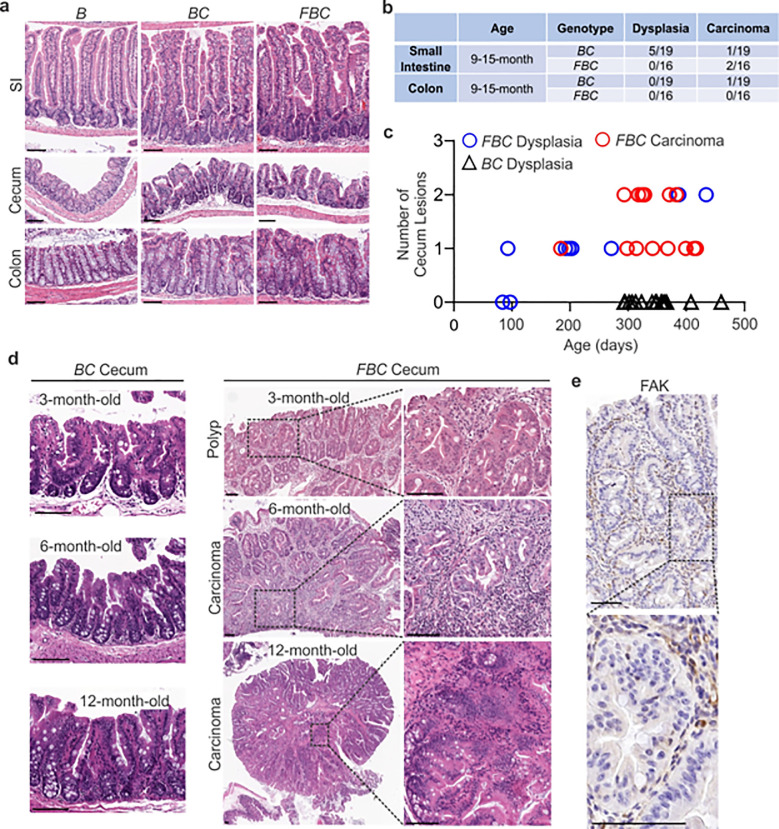
*Fak* loss enhances *BRAF*^V600E^-driven cecal tumorigenesis in mice. **a** Representative hematoxylin and eosin (H&E) staining of the small intestine, cecum, and colon from indicated 6-week-old mice. **b** Summary of tumor incidence at small intestine and colon in indicted mice at the indicated age. **c** Summary of tumor incidence and tumor stage at cecum in indicated mice at the indicated age. **d** H&E staining of the cecum in *BC* mice and cecal serrated adenoma/polyp and carcinoma in *FBC* mice at the indicated age. **e** Representative IHC staining of Fak in cecal tumors in *FBC* mice. Scale bars: 100 μm.

Similar to that seen in *BC* mice, compared to the *BRAF*^V600E/+^ (B) mice, the *FBC* mice exhibited hyperplasia throughout the intestine (Fig. 2a) and thickened small and large intestines (Supplementary Fig. 1c). In *BC* mice, intestinal tumors were primarily developed in the small intestine at nine months or older (Fig. 2b). *Fak* loss had minimal impact on tumor incidence in the small intestine and the colon; however, it greatly enhanced BRAF^V600E^-induced cecal tumor formation: cecal tumor incidence increased from 0% (0/15) in 9-month or older *BC* mice to 100% (16/16) in *FBC* mice (Fig. 2c). Cecal adenoma/polyp started to develop in 3-month *FBC* mice and after six months, all mice (4/4) developed cecal tumors and 25% of the tumors (1/4) were carcinomas (Fig. 2c, d). At nine months or older, 100% of the mice developed cecal tumors with a high incidence (13/16) of carcinoma (Fig. 2c, d, and Supplementary Fig. 1d). IHC staining confirmed that while the stroma showed strong Fak staining, tumor cells were Fak negative (Fig. 2e), hence validating that tumors were originated from Fak-deleted epithelial cells. Of note, no tumor metastasis was found in *FBC* mice. *FBC* mice were aged up to 434 days, and the life span of *FBC* mice was similar to that of BC mice.

Together, these results revealed that *Fak* deletion promotes, rather than inhibits, *BRAF*^V600E^-induced cecal tumor formation. *BRAF*-mutant CRCs are primarily located in the right colon, including the cecum ^30^. The same primary tumor location suggests that the *FBC* model truthfully recapitulates human *BRAF*-mutant serrated CRCs, at least by location.

### The molecular feature of the cecal tumors in *FBC* mice closely resembles human SSA/Ps.

**Figure 3:**
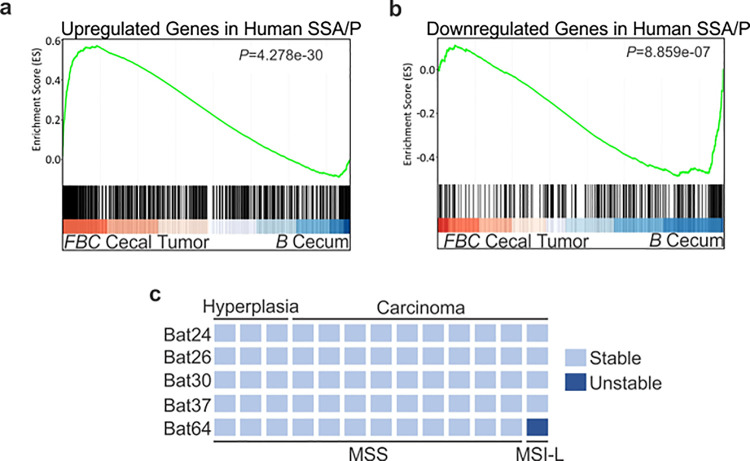
Molecular characterization of cecal tumors in *FBC* mice. **a** GSEA plot showing enrichment of human SSA/Ps signature genes (upregulated genes in SSA/Ps) in *FBC* cecal tumors vs normal cecal mucosa of *B* mice. **b** GSEA plot showing that downregulated genes in human SSA/Ps were also reduced in *FBC* cecal tumors. **c** Microsatellite instability status of *FBC* mice cecal mucosa and cecal carcinomas. Each column represents one sample.

To characterize the molecular signatures of the cecal tumor in *FBC* mice, we performed whole-exome sequencing on paired tumors (n=2) and neighboring mucosa. No additional driver mutations were detected in the cecal tumors (Supplementary Table 1), implying that cecal tumor formation in *FBC* mice does not require additional driver mutations. To evaluate the relevance of *FBC* cecal tumors to humans, we performed RNA-sequencing (RNA-seq) and Gene Set Enrichment Analysis (GSEA) to determine whether *FBC* cecal tumors exhibited similar gene expression signatures as human SSA/Ps ^31^. The results showed that upregulated genes in human SSA/Ps were significantly enriched in cecal tumors in *FBC* mice (Fig. 3a). Downregulated genes in human SSA/P were also reduced in *FBC* tumors (Fig. 3b). Together, these results suggest that the *FBC* cecal tumors greatly resemble human serrated lesions at the molecular level.

About 50% of *BRAF*-mutated CRCs exhibit defective DNA mismatch repair ^18^. The results of microsatellite instability (MSI) analysis indicated that most *FBC* cecal tumors were microsatellite stable (MSS) (Fig. 3c). It has been shown that mismatch repair deficiency accelerates *BRAF*-driven serrated tumorigenesis ^32^. Maximizing the oncogenic activity of BRAF^V600E^ without mismatch repair gene mutation and additional driver mutations suggests that in *FBC* mice, Fak loss created a “just-right” environment optimal for MSS serrated cecal tumor to form.

### Fak loss increases intestinal stemness by upregulating Lgr4 levels in *FBC* mice.

We explored the molecular mechanism underlying *Fak* loss-enhanced cecal tumor formation. Consistent with a prior report ^27^, we did not detect any abnormalities in the intestine in *Vill-Cre*; *Fak*^fl/fl^ mice, implying that FAK loss by itself is not a driving force for intestinal tumorigenesis. A prior study showed that upon TGFβ (transforming growth factor β) receptor inactivation, *BRAF*^V600E^-induced right-sided tumorigenesis is supported by microbial-driven inflammation ^33^. To test the role of inflammation in *FBC* tumor formation, we compared sub-cryptal proprial neutrophil infiltration using myeloperoxidase (MPO) as a neutrophil marker for IHC staining. The results showed that, consistent with prior findings ^33^, the number of MPO-positive cells was significantly higher in *BC* mice than in *B* mice; however, Fak loss did not further increase neutrophil infiltration in FBC mice (Supplementary Fig. 2a). Consistent with this, GSEA results showed that there was no difference in the expression of inflammatory response genes ^34^ in *FBC* mice and *BC* mice (Supplementary Fig. 2b). Together, these findings imply that Fak loss promotes tumor formation not by enhancing intestinal inflammation.

We next evaluated the roles of cellular senescence, apoptosis, cell proliferation, and *Lgr5* expression in cecal tumorigenesis in *FBC* mice. The results indicated that BRAF^V600E^ was insufficient to trigger senescence evaluated by SA-β-galactosidase staining or apoptosis evaluated by the TUNEL staining in *BC* mice (Supplementary Fig. 2c, d). Bromodeoxyuridine (BrdU) incorporation assays confirmed mutant BRAF-induced hyperproliferation. However, Fak loss did not further enhance the BrdU incorporation rate (Supplementary Fig. 2e). These results indicated that *Fak* deletion promotes tumor formation not through modulating cellular senescence, apoptosis, and cell proliferation.

**Figure 4:**
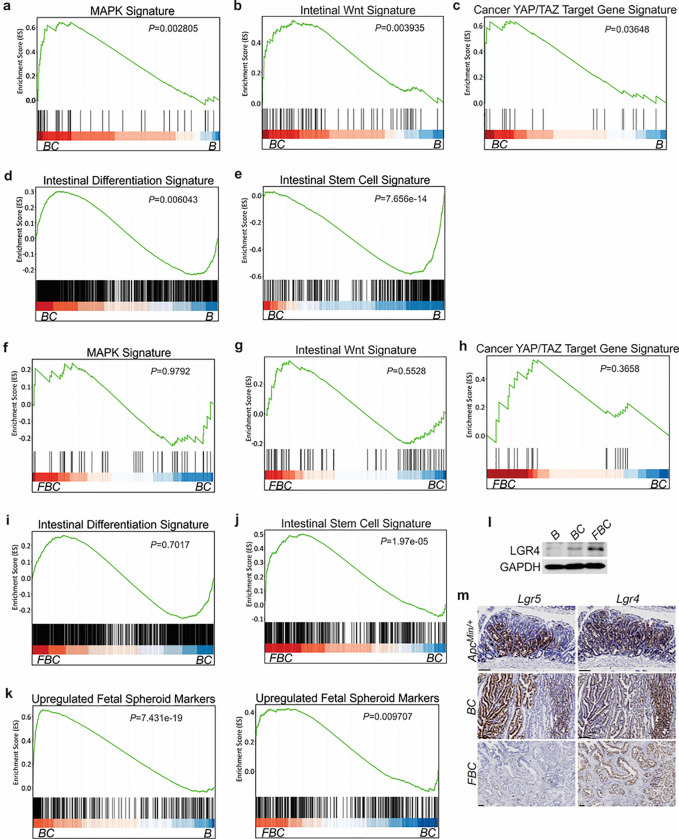
*BRAF*^V600E^ mutation and *Fak* loss-mediated changes in signaling pathways. GSEA analysis showing upregulation of MAPK signature (**a**), intestinal WNT signaling (**b**), YAP/TAZ target gene signature (**c**) and intestinal differentiation signature (**d**), and downregulation of intestinal stem cell signature (**e**) in the cecum of *BC* mice vs *B* mice (n=4 per group). GSEA plots revealed no significant change in MAPK signature (**f**), intestinal WNT signaling (**g**), YAP/TAZ target gene signature (**h**), and intestinal differentiation signature **(i**) in the cecum of *FBC* mice vs *BC* mice, but enrichment of stem cell signature in *FBC* mice (**j**) (n=4 per group). **k** GSEA analysis showing upregulation of upregulated fetal spheroid markers in the cecum of *BC* mice vs *B* mice, and further enrichment in the cecum of *FBC* mice vs *BC* mice (n=4 per group). **l** Immunoblotting analysis of LGR4 in the cecum from indicated 6-week-old mice. **m** Representative in situ hybridization (ISH) staining of tumor sections from *Apc*^*Min/+*^*, BC*, and *FBC* mice using *Lgr4* and *Lgr5* probes. Scale bars: 100 μm.

Given that BRAF^V600E^ drives tumorigenesis through constitutive downstream ERK1/2 activation ^13^, we examined the impact of Fak loss on ERK pathway transcriptional output. GSEA analysis showed that ERK pathway output was significantly increased in *BC* mice (Fig. 4a), which was consistent with the earlier report ^20^, but Fak loss did not further enhance it (Fig. 4f). Wnt pathway activation ^32^ and activation of transcription co-factor YAP have been implied in BRAF^V600E^-induced serrated tumorigenesis ^33^. In this study, our GSEA results also showed that the expression of intestinal Wnt signature genes ^35^ and YAP target genes ^36^ were significantly higher in *BC* mice than in *B* mice (Fig. 2b, c). Again, Fak loss did not further enhance the activations (Fig. 2g, h). Together, these findings excluded the possibility that Fak loss promotes cecal tumor formation by enhancing ERK pathway output and activation of the Wnt and YAP pathways.

BRAF^V600E^ poorly initiates colon cancer in mice due to oncogenic BRAF-induced tissue differentiation and loss of intestinal stem cells ^15^. With this, GSEA results showed increased expressions of intestinal differentiation signature genes ^37^ (Fig. 4d) and decreased expressions of intestinal stem cell signature genes ^38^ (Fig. 4e) in *BC* mice. Fak deletion did not reverse BRAF^V600E^-induced tissue differentiation (Fig. 4i) but significantly enhanced intestinal stemness (Fig. 4j). These results revealed that Fak deletion promotes BRAF^V600E^-induced cecal tumor formation through increasing intestinal stemness.

The adult stem cell marker Lgr5 and its relative Lgr4 are R-spondin receptors mediating R-spondin signaling and are critical for intestinal stemness ^39,40^. Mutant BRAF reduces *Lgr5* expression in the intestinal crypt ^15,33^. Our results confirmed the downregulation of *Lgr5* in the cecum crypt in *BC* mice, and we found that Fak loss did not restore *Lgr5* expression in *FBC* mice (Supplementary Fig. 2f). These results thus excluded the possibility that Lgr5 mediates Fak loss-induced intestinal stemness.

Prior studies show that the fetal type of intestinal stem cells has a strikingly different transcriptome than that of adult intestinal stem cells, and the receptor LGR4, but not LGR5, is essential for the cells ^41^. In VillinCre^ER^;*Braf*^LSL-V600E/+^;*Alk5*^fl/fl^ mice, the proximal colonic tumors exhibit fetal intestinal signature ^33^. Consistent with the notion that mutant *BRAF*-driven right-sided colonic tumors are fetal progenitor phenotypes, GSEA results confirmed enrichment of the fetal-type transcriptomic signatures ^41^ in cecal mucosa in *BC* mice. The fetal signature was further enriched in FBC mice (Fig. 4k). Accordingly, the immunoblotting analysis showed that the protein level of Lgr4 was increased in the intestine epithelium in FBC mice (Fig. 4i). Consistent with the fact that intestinal Lgr5 expression was low in FBC mice (Supplementary Fig.2f), FBC tumors mainly expressed Lgr4 but not Lgr5. In contrast, *BC* and *Apc*^min/+^ tumors expressed both Lgr5 and Lgr4 (Fig. 4m). These results suggest that upregulated Lgr4 mediated the intestinal stemness increase in *FBC* mice.

### FAK loss downregulates EGFR-dependent ERK phosphorylation to increase Lgr4 mRNA expression and protein stability.

**Figure 5:**
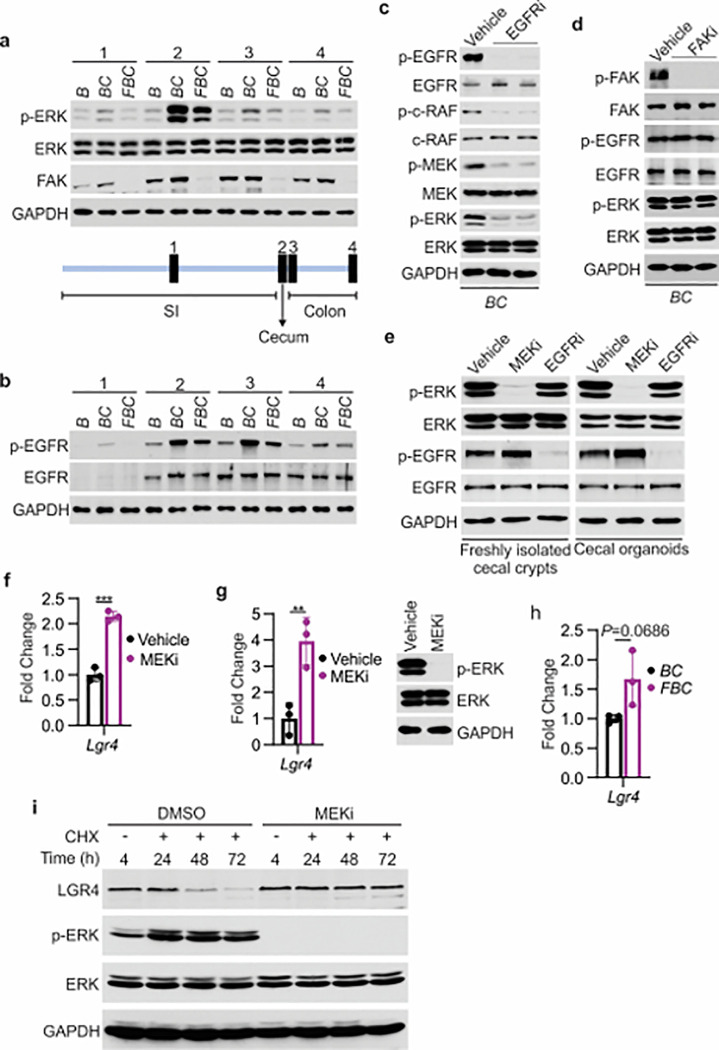
Fak loss inhibits ERK phosphorylation and upregulates Lgr4. **a** and **b** Immunoblotting analysis of intestinal mucosa lysates from indicated bowel subsites in indicated 6-week-old mice. **c** Immunoblotting analysis of cecum lysates from 6-week-old *BC* mice treated with vehicle or EGFR inhibitor erlotinib for 4 hours. Each lane represented a single mouse. **d** Immunoblotting analysis of cecum lysates from 6-week-old *BC* mice treated with vehicle or FAK inhibitor PF-562271 for 4 hours. Each lane represented a single mouse.**e** Immunoblotting analysis of lysates from freshly isolated cecal crypts and cecal organoids treated with DMSO, MEK inhibitor PD0325901, or erlotinib, respectively as described in Method. **f**qRT-PCR of *Lgr4* using lysates from HT-29 cells treated with the vehicle and MEKi for 4 hours. Data presented as mean ± SD (***P<0.001; Student’s *t*-test, two-tailed). **g** qRT-PCR of *Lgr4* using cecum lysates from *BC* mice treated with vehicle or MEKi for 6 hours. Data presented as mean ± SD (**P<0.01; Student’s *t*-test, two-tailed). Abrogation of ERK phosphorylation at T202/Y204 in the cecum was confirmed by western blot. **h** qRT-PCR of *Lgr4* in cecum from *BC* and *FBC* mice (n=3 per group). Data presented as mean ± SD (*P* value calculated using two-tailed Student’s *t*-test). **i** Immunoblotting analysis of the lysates from HT-29 cells treated with cycloheximide (100 μg/ml) and/or MEK inhibitor PD0325901 (10 μM) as indicated.

We addressed how Fak loss mediates Lgr4 increase. A prior study suggested that Wnt signaling maintains quiescent intestinal stem cell pools through suppression of the MAPK pathway in the intestine ^42^. Given the fact that Fak loss did not jeopardize ERK pathway transcriptional output (Fig. 4f), Fak loss may increase intestinal stemness by inhibiting ERK phosphorylation. To test, we first compared the levels of phosphorylated ERK across the intestines in *B* mice, *BC* mice, and *FBC* mice. As anticipated, BRAF^V600E^ increased p-ERK levels throughout the intestine (Fig. 5a). FAK is positively involved in ERK1/2 activation ^24^. Consistent with this, in *FBC* mice, FAK deletion suppressed mutant BRAF-induced elevation of p-ERK (Fig. 5a). The decoupling of ERK pathway output (no change) and the level of p-ERK (reduced) upon Fak loss is in line with a prior report suggesting that the level of ERK phosphorylation does not truthfully reflect ERK pathway activation ^20^.

We next examined how Fak loss altered BRAF^V600E^-induced phosphorylation of ERK. A prior study found that FAK promotes EGFR signaling ^43^, raising the possibility that FAK regulates ERK phosphorylation through EGFR. We then evaluated Egfr activation (represented by phosphorylated EGFR at tyrosine 1068) in the mice. The results showed that the level of phosphorylated Egfr^Y1068^ was increased in *BC* mice throughout the intestine (Fig. 5b). In *FBC* mice, Fak deletion moderately reduced BRAF^V600E^-induced Egfr activation (Fig. 5b) and suppressed Egfr downstream signal transduction as evidenced by the decreased levels of phosphorylated c-Raf^S338^ and MEK1/2^S217/221^ in *FBC* mice (Supplementary Fig. 3a). To validate that EGFR indeed regulates BRAF^V600E^-induced ERK phosphorylation, we treated *BC* mice with the EGFR inhibitor erlotinib. Erlotinib treatment, without significantly reducing ERK pathway output (Supplementary Fig. 3b), indeed suppressed phosphorylation of C-RAF, MEK, and ERK (Fig. 5c). Of note, Fak deletion had no impact on the level of p-EGFR and p-ERK in control mice (Supplementary Fig. 3c). Inhibition of Fak kinase activity by FAK inhibitor PF-562271 did not affect the phosphorylation of Egfr and ERK (Fig. 5d), implying that the kinase activity of Fak is not involved in the FAK/EGFR/ERK regulation in BRAF^V600E^-induced serrated tumorigenesis.

FAK complexes with activated EGFR to promote EGFR signaling ^43^. We assessed whether FAK interacts with EGFR in *BRAF*^V600E^-mutant cells. The results of co-immunoprecipitation using lysates from cecal mucosa confirmed the Fak-Egfr interaction and revealed that the Fak-Egfr interaction was increased in *BC* mice and inhibition of Egfr appeared not to affect the Fak-Egfr binding (Supplementary Fig. 3d). ERK phosphorylation is refractory to EGFR inhibition in human BRAF^V600E^-mutant CRC cell lines ^22,23^, however, the FAK-EGFR interaction was still detected in HT29 CRC cells and the interaction was not affected by either EGFR inhibition or FAK inhibition (Supplementary Fig. 3e). These results indicated that FAK/EGFR interaction alone is not sufficient for FAK getting involved in the regulation of MAPK signaling.

The contradictory results seen in *BC* mice and human *BRAF*^V600E^-mutant CRC cell lines could result from the differences between *in vitro* culture systems and *in vivo*. To test, we examined whether inhibition of Egfr leads to ERK inhibition in freshly isolated cecal crypts from *BC* mice and *BC* cecal organoids. The results showed that inhibition of Egfr did not reduce ERK phosphorylation, confirming that the contradictory findings resulted from *in vitro* and *in vivo*. We speculate that the lack of certain stromal factors *in vitro* is responsible for the EGFR’s inability to transmit its signal to activate ERK.

Finally, we examined whether and how a reduction in ERK phosphorylation increases Lgr4 expression/stemness. Our results showed that treatment with MEK inhibitor increased the mRNA expression of LGR4 in human *BRAF*^V600E^-mutant CRC HT29 cells (Fig 5f) and *BC* mice (Fig. 5g), uncovering a negative association between the level of ERK phosphorylation and mRNA expression of Lgr4. Of note, inhibition of ERK activation in *BC* mice was confirmed by the abrogation of ERK phosphorylation (Fig 5g) and suppression of ERK pathway transcriptional output (Supplementary Fig. 4). This negative association was further supported by our observation that the mRNA levels of Lgr4 were higher, albeit not statistically significant, in *FBC* mice than in *BC* mice (Fig 5h). Regulation of Lgr4 protein stability represents an important mechanism of modulating Lgr4 function ^44^. Our cycloheximide chase analysis results showed that inhibition of ERK phosphorylation by MEK inhibitor treatment dramatically enhanced Lgr4 protein stability in *BRAF*^V600E^-mutant CRC cell line HT29 cells (Fig 5i). This finding revealed the inverse correlation between the level of ERK phosphorylation and the protein stability of Lgr4. These results suggest that Fak loss lowers BRAF^V600E^-induced ERK phosphorylation to increase Lgr4 mRNA expression and protein stability, thereby enhancing intestinal stemness and cecal tumor formation.

### Inhibition of ERK phosphorylation downregulates the level of E3 ubiquitin ligase NEDD4.

**Figure 6:**
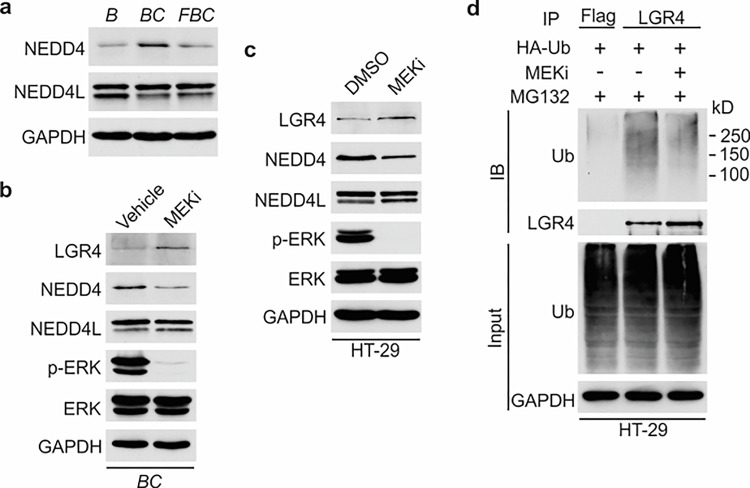
Inhibition of ERK phosphorylation stabilizes LGR4 through downregulating NEDD4. **a** Immunoblotting analysis of cecum lysates from indicated 6-week-old mice. **b**Immunoblotting analysis of cecum lysates from 6-week-old *BC* mice treated with vehicle or MEK inhibitor PD0325901. MEK inhibitor was given to the mice at a dose of 25 mg/kg three times at 12 hours intervals. Twenty-eight hours after the first treatment, the cecum mucosa was collected for immunoblotting. **c**Immunoblotting analysis of lysates from HT-29 cells treated with DMSO or 10 μM MEK inhibitor for 24 hours. **d** HT-29 cells were transfected with HA-Ubiquitin. One day later, the cells were treated with DMSO or 10 μM MEK inhibitor for 24 hours. Then all the cells were incubated with 10 μM MG132 for additional 4 hours. The cell lysates were collected for immunoprecipitation and immunoblotting with the indicated antibodies.

We next investigated how the reduction of ERK phosphorylation increases Lgr4 stability. The HECT-domain E3 ligases NEDD4 (Neuronal precursor cell developmentally downregulated protein 4) and its homolog NEDD4L can ubiquitinate Lgr4, leading to its degradation ^45^. Although the RNA-seq data showed no difference in mRNA expression levels of Nedd4 and Nedd4l in *C57, BC*, and *FBC* mice, the protein level of Nedd4, but not Nedd4l, was increased in *BC* mice then decreased in *FBC* mice (Fig. 6a). To confirm that loss of ERK phosphorylation mediates the Nedd4 reduction, we treated the *BC* mice with MEK inhibitor and measured the protein levels of Nedd4 and Nedd4l. As shown in Fig. 6b, MEK inhibitor treatment abrogated ERK phosphorylation and reduced the expression of Nedd4, accompanied by increased Lgr4 level. These data suggested that reduced ERK phosphorylation reduces E3 ligase Nedd4 to increase Lgr4 stability. The decreased ubiquitination of LGR4 was confirmed in HT-29 cells. While treatment with MEK inhibitor inhibited the expression of NEDD4 (Fig. 6c), it greatly reduced the ubiquitination of LGR4 (Fig 6d). Together, these data implied that reduction in ERK phosphorylation reduces the expression of E3 ubiquitin ligase Nedd4 in *FBC* mice to increase the Lgr4 level.

### FAK’s influence on oncogenic MAPK-driven intestinal tumorigenesis depends on FAK’s impact on ERK phosphorylation.

**Figure 7:**
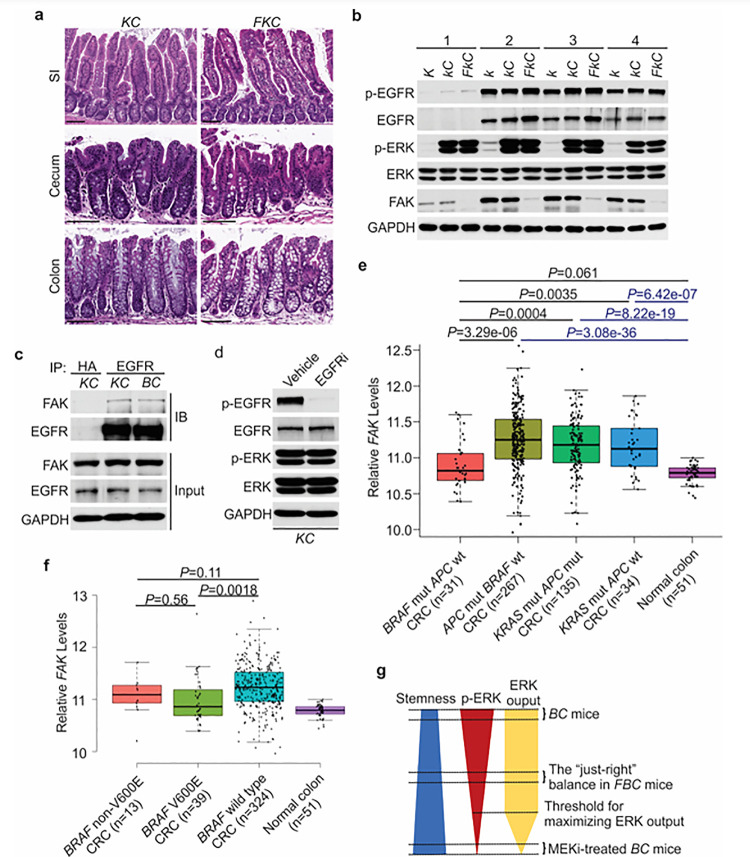
ERK activation is FAK/EGFR-independent in *KC* mice. Representative hematoxylin and eosin (H&E) staining of the small intestine, cecum, and colon from indicated 9-month-old mice. **b** Immunoblotting analysis of intestinal mucosa lysates from indicated bowel subsites in indicated 6-week-old mice. **c**The cecal mucosa lysates from 6-week-old *KC* and *BC* mice were used for immunoprecipitation and immunoblotting with the indicated antibodies. **d** Immunoblotting analysis of cecum lysates from 6-week-old *KC* mice treated with vehicle or EGFR inhibitor erlotinib for 4 hours. **e** and **f** Comparison of *FAK*expression levels between CRCs with indicated mutations by analysis of TCGA RNA-sequencing dataset. Data were analyzed for statistical significance using a Student t-test. **g** Diagram of the “just-right” MAPK signaling model in the serrated pathway.

Fak loss reduced ERK phosphorylation in *FBC* mice (Fig. 5a) but not in control mice with wild-type *BRAF* (Supplementary Fig. 3c). To determine whether FAK is involved in other oncogenic MAPK-driven tumors, we generated *Vill-Cre*;*Kras*^LSL-G12D/+^ (*KC*) mice and *Vill-Cre*;*Kras*^LSL-G12D/+^;*Fak*^fl/fl^ (*FKC*) mice. In *KC* mice, the endogenous expression of oncogenic Kras induces serrated hyperplasia; however, high ERK activation-induced senescence prevents hyperplasia progression into dysplasia ^46^. As shown in Fig. 7a, no tumor was found in *KC* mice (n=6, 9-months-old) and *FKC* mice (3-month-old, n=3; 6-month-old, n=3; 9-month-old, n=4). Immunoblotting results confirmed that Fak loss failed to influence the phosphorylation of Egfr or ERK (Fig. 7b). The co-immunoprecipitation results showed that Fak complexed with Egfr in *KC* mice similarly as in *BC* mice (Fig.7c), implying that the noninvolvement of Fak was not due to the lack of Fak/Egfr interaction. A recent preprint (https://doi.org/10.1101/2020.07.02.185173) suggests that “EGFR network oncogenesis cooperates with weak oncogenes in the MAPK pathway”, which inspired us to propose the notion that EGFR participates in the regulation of ERK phosphorylation only when the p-ERK level is relatively low. In *KC* mice, KRAS^G12D^ induces extremely high levels of ERK phosphorylation, high enough to cause intestinal senescence ^46^. Given the level of increased p-ERK in *KC* mice, one would expect that ERK phosphorylation is EGFR-independent. The EGFR independence was confirmed by our results showing that pharmacologic abrogation of EGFR activation had no impact on KRAS^G12D^-induced ERK phosphorylation in *KC* mice (Fig 7d). Clinical findings further supported our notion. Anti-EGFR therapy is excluded for patients with *KRAS*-mutant CRC, supporting that EGFR has minimum impact on downstream MAPK signaling upon *KRAS* mutation. However, when ERK activation is inhibited by KRAS^G12C^ inhibitors, EGFR signaling acts as the dominant mechanism of colorectal cancer resistance to KRAS^G12C^ inhibitors ^47^.

To address whether FAK downregulation is specific to human *BRAF*-mutant CRCs, we compared FAK expression levels in CRCs with different driver mutations using the TCGA database. TCGA analysis revealed that *FAK* mRNA levels were significantly lower in *BRAF*-mutated CRCs than in *APC*-mutated CRCs or *KRAS*-mutant CRCs (Fig. 7e). This result is consistent with the result seen in mice, again, it suggests that FAK is not involved in the regulation of *KRAS*-mutant CRCs.

In mice, mutant BRAF-induced ERK activation is cancer stage-dependent with significantly higher levels of phosphorylated ERK in high-grade dysplasia and carcinoma ^3^, suggesting that different tumor stages may require different levels of p-ERK. If FAK is a key regulator of ERK phosphorylation in mutant *BRAF*-induced serrated tumorigenesis in patients, one would expect the level of FAK may increase as the tumors progress. Consistent with this notion, we observed that FAK levels were higher in BRAF-mutant CRCs than in BRAF-mutant polyps (Fig 1a), TCGA analysis (Fig. 7e) further confirmed that FAK expression was restored to a level similar to normal intestines, albeit still significantly lower than in APC mutant or KRAS mutant CRCs (Fig. 7e).

In patients, BRAF mutations are divided into two groups: Activator and amplifier mutation ^48^. In CRC, the majority (80%–90%) of activating mutations in BRAF are V600E ^18^. Among these mutants, based on their kinase activities, BRAF^V600E^ belongs to the high-activity mutants, and the rest of the mutants except G595R (with impaired BRAF kinase activity *in vitro* but still induce constitutive ERK activation *in vivo*) are intermediate activity mutants ^49^. If mutant BRAF-induced ERK phosphorylation needs to reach a “just-right” level via FAK downregulation in patients, one would expect that the degree of FAK downregulation is BRAF mutant activity-dependent, and there could be a correlation between the activity of BRAF mutants and the degree of FAK reduction. Consistent with this speculation, TCGA data analysis confirmed that CRCs with *BRAF*^V600E^ mutation had lower FAK expression than CRCs with non-V600E mutations and *BRAF* wild-type CRCs (Fig. 7f). Although the differences between V600E and non-V600E groups were not statistically significant due to limited sample numbers, they might be biologically relevant.

## Discussion

The current study finds that in *BRAF*^V600E^-mutant intestinal epithelium, elevating the p-ERK level to a minimum threshold is sufficient to maximize the pathway transcriptional output, i.e., only lowering the p-ERK level below the threshold will significantly abrogate the ERK pathway transcriptional output. Due to the negative association between ERK phosphorylation and intestinal stemness, any increase in ERK phosphorylation will decrease intestinal stemness (Fig 6g). In *BRAF*^V600E^-mutant intestinal epithelium, ERK phosphorylation is EGFR/RAS/c-RAF-dependent. The involvement of EGFR provides an opportunity for non-MAPK pathway factors such as FAK to participate in the regulation of ERK phosphorylation to influence the biological outcomes of *BRAF* mutation. This study has established the first “just-right” MAPK signaling model of BRAF^V600E^-induced tumor formation (Fig. 7g). Our results show that by lowering BRAF^V600E^-induced ERK phosphorylation, Fak loss, without jeopardizing the ERK pathway transcriptional output, enhances mRNA expression and protein stability of Lgr4, thereby increasing intestinal stemness and promoting cecal tumor formation in mice.

High-level activation of oncogenes (e.g., KRAS, BRAF, and c-MYC) triggers intrinsic tumor suppression ^46,50–53^. Genetic abrogation of tumor suppressors such as p53 or p16 revokes the tumor-suppressive barrier, thereby facilitating oncogene-induced tumorigenesis ^4,46,51,52^. Cooperation with other oncogenic stimulation, such as co-expression of c-MYC and KRAS, ultraviolet radiation on melanocytes expressing BRAF^V600E^, can also break the suppressive barrier ^54,55^. In cellular models ^56,57^, overexpression of MKP/DUSPs evades high ERK activation-induced tumor suppression. Whether and how the suppressive barrier can be avoided or reduced *in vivo* has never been experimentally tested. The current study is the first demonstration that mutant BRAF-induced activation of ERK signaling is tuneable *in vivo*, and by tuning ERK activation to alter the suppressive barrier, FAK regulates BRAF transforming activity.

In *BRAF*-mutated melanoma, a complete shutdown of the MAPK pathway is necessary for significant tumor response ^58^. In patients with *BRAF*^V600E^-mutated CRCs, a combination of encorafenib, cetuximab, and binimetinib (MEK inhibitor) treatment increased the response rate to 26% ^59^, highlighting the importance of complete ERK pathway inhibition. However, the inverse correlation between the level of phosphorylated ERK and the level of stemness/Lgr4 expression seen in mutant BRAF expressing intestinal epithelial cells let us speculate that inhibition of ERK phosphorylation may cause stemness increases in *BRAF*-mutated CRC cells. The molecular mechanisms underlying ERK phosphorylation inhibition-mediated stemness increase remain to be determined. Given the importance of cancer cell stemness in treatment resistance ^60^, we propose that the optimal treatment outcome can only be achieved when the inhibition of ERK phosphorylation-mediated stemness increase is simultaneously suppressed.

In sum, the current study reveals the existence of a balance—between the level of phosphorylated ERK, the level of ERK pathway output, and the level of intestinal stemness. Our results show that the “just-right” balance optimal for *BRAF*^V600E^-induced cecal tumor formation can be achieved through FAK alteration. Achieving optimal treatment response in *BRAF*-mutated CRC patients, though, may require abrogation of the p-ERK-stemness regulatory link. That said, the current study could have profound implications for the development of new anticancer agents and new treatment approaches for patients with *BRAF*-mutated CRC.

## Methods

### Mice and Treatment.

All animal procedures were performed according to protocols approved by the Institutional Animal Care and Use Committee at the University of Pittsburgh. Mice were fed a standard diet (diet ID 5P75; Purina LabDiet, St. Louis, MO). *Fak*^*fl/fl*^ mice were received from the Mutant Mouse Resource & Research Centers (MMRRC, cat. no. 009967-UCD). *Villin-Cre* (cat. no. 021504), *Braf*^*LSL-V600E/+*^ (cat. no. 017837), *Kras*^*LSL-G12D/+*^ (cat. no. 008179) and *Rosa26-tdTomato* (cat. no. 007914) mice were obtained from the Jackson Laboratory. Genotyping was performed according to the protocols provided by MMRRC and the Jackson Laboratory. *Villin-Cre* and *Braf*^*LSL-V600E/+*^ mice were crossed to get the *BC* mice. The littermates harboring *Braf*^*LSL-V600E*^ allele were used as controls whenever available. To get the *FBC* mice, *Fak*^*fl/fl*^ mice were first crossed with *Villin-Cre* mice and *Braf*^*LSL-V600E/+*^ mice, respectively. The offspring *Villin-Cre;Fak*^*fl/+*^ and *Braf*^*LSL-V600E/+*^*;Fak*^*fl/+*^ mice were further crossed with *Fak*^*fl/fl*^ mice to get the *Villin-Cre;Fak*^*fl/fl*^ (*FC*) and *Braf*^*LSL-V600E/+*^*;Fak*^*fl/fl*^ (*FB*) mice. The *FBC* mice were finally obtained by crossing *FC* and *FB* mice. The same strategy was used to generate the *FKC* mice. *BC, FBC, KC* and *FKC* mice were euthanized at the indicated age to evaluate the tumor formation. *Villin-Cre* mice and *Rosa26*^*LSL-tdTomato/LSL-tdTomato*^ mice were crossed to get the *Villin-Cre*; *Rosa26*^*LSL-tdTomato/+*^ mice.

For Bromodeoxyuridine (BrdU) labeling, six-week-old mice were given BrdU (MilliporeSigma) at a dose of 100 mg/kg by intraperitoneal injection two hours prior to harvesting. For inhibitor treatment, six-week-old mice were given vehicle (a mixture of 50% DMSO and 50% PEG 400), PF-562271 (60 mg/kg in vehicle) or Erlotinib (100 mg/kg in the vehicle) by a single oral gavage four hours (for immunoblotting) or six hours (for qRT-PCR analysis of ERK output genes) before harvesting. MEK inhibitor PD0325901 was given to mice by oral gavage at a dose of 25 mg/kg in the vehicle. All experiments were performed in both male and female mice.

Plasmid and transient transfection. pcDNA3-HA-Ubiquitin (18712) was from Addgene. Plasmid transient transfections were performed using PolyJet In Vitro DNA Transfection Reagent (SignaGen) according to the manufacturer’s instructions.

### Cell culture and treatment.

HT-29 cells were obtained from the American Type Culture Collection (ATCC) and cultured in DMEM supplemented with 5% fetal bovine serum, 100 units/ml penicillin and 100 μg/ml streptomycin, in a 37°C humidified incubator containing 5% CO2. To study the interaction between FAK and EGFR in HT-29 cells, the cells were treated with DMSO, PF-562271 (5 μM) or erlotinib (10 μM) for one hour before harvested for immunoprecipitation. To study the ubiquitination of LGR4, HT-29 cells were treated with DMSO or 10 μM MEK inhibitor PD0325901 for 24 hours. Then 10 μM MG132 was added to the culture medium and incubated for additional 4 hours before harvesting the cells for immunoprecipitation.

### Protein stability assay.

HT-29 cells were seeded twenty-four hours before the experiments. The cells were treated with 100 μg/ml cycloheximide (Selleck Chemicals), 10 μM MEK inhibitor PD0325901, or their combination as indicated. Then the cells were harvested, and the whole cell lysates were used for immunoblotting.

### Organoid culture and treatment.

Mouse organoids were isolated according to the published protocol with some modifications ^61^. Briefly, the cecum of the *BC* mouse was rinsed with cold PBS, cut into small pieces, and washed eight times in cold PBS by gently pipetting. The fragments were incubated in 10 mM EDTA diluted in PBS for 8 minutes in a 37 °C tube rocker. Then the EDTA solution was removed and the tissue was pipetted 10 times in cold PBS. The supernatant was collected and centrifuged at 300 × g for 3 minutes at 4 °C. The cell pellet was washed with DMEM/F-12 medium and centrifuged at 400 × g for 3 minutes at 4 °C. The pellet was resuspended in Cultrex Reduced Growth Factor Basement Membrane Extract, Type R1 (R&D Systems), and seeded into a 24-well plate. Organoids were cultured using Mouse IntestiCult^™^ Organoid Growth Medium (STEMCELL Technologies) in a 37°C humidified incubator containing 5% CO_2_. The medium was changed every other day. For inhibitor experiments, the freshly isolated crypts (one hour after seeding) and organoids (five days after seeding) were treated with 10 μM EGFR inhibitor erlotinib and 10 μM MEK inhibitor PD0325901, respectively, for two hours. To isolate protein for immunoblotting after treatment, the crypt cultures were scraped and suspended in 500 μl of TrypLE Express containing 10 μM EGFR inhibitor or 10 μM MEK inhibitor and incubated at a 37°C water bath for 5 minutes with occasional agitation. After the addition of 500 μl of DMEM/F-12 medium, the crypt cultures were centrifuged at 400 × g for 3 minutes at 4 °C. The cell pellets were resuspended in cold PBS and centrifuged again. The final pellets were lysed in RIPA buffer (Alfa Aesar) supplemented with protease inhibitor and phosphatase inhibitor (Thermo Fisher Scientific). Crypt cultures treated with DMSO were used as controls. The lysates were quantified and resolved by sodium dodecyl sulfate-polyacrylamide gel electrophoresis (SDS-PAGE) and blotted with the indicated antibodies.

### Immunoblotting and immunoprecipitation.

After the mice were euthanized, the entire intestines were immediately removed and rinsed twice with ice-cold PBS. The mucosal layers of the small intestine (about 1 cm length), colon (about 1 cm length), and cecum (entire cecum, without appendix) were harvested by scraping with a blade and all procedures were performed on ice. The freshly collected tissue was lysed in RIPA buffer supplemented with protease inhibitor and phosphatase inhibitor. The lysates were quantified and resolved by SDS-PAGE and blotted with the indicated antibodies. SuperSignal Western Blot Enhancer (Thermo Fisher Scientific) was used to enhance the blotting signal when needed. To detect the interaction between FAK and EGFR, the tissue lysates were pre-cleared with Protein G-sepharose beads at 4°C for 30 min. The cleared lysates were incubated with anti-EGFR antibody conjugated to agarose (Santa Cruz Biotechnology) or anti-HA affinity gel (MilloporeSigma) at 4°C for 4 hr. The immunoprecipitates were washed three times with lysis buffer containing 20 mM Tris-HCl, pH 7.5, 150 mM NaCl, 1 mM EDTA, 1% NP40, and 10% Glycerol, and subjected to SDS-PAGE followed by immunoblotting. The same protocol was used for immunoprecipitation experiments with HT-29 cell lysates. The cell lysates precipitated with anti-HA or anti-Flag beads were used as controls. The antibodies used for immunoblotting are shown in Supplementary Table 2. All experiments were independently repeated at least three times.

### Immunohistochemistry, in situ hybridization, BrdU staining, TUNEL staining, and histopathology.

The de-identified human colon tissue samples from *BRAF*^V600E^-mutated CRC patients were provided by the University of Pittsburgh School of Medicine, Department of Pathology tissue core. For mouse tissue sections, the mouse intestine was dissected out, rinsed twice with ice-cold PBS, fixed overnight in 10% neutral buffered formalin at 4°C, embedded in paraffin, and finally cut into 5-μm sections. The sections were deparaffinized in xylenes and rehydrated in graded alcohol solutions, followed by washes in distilled water. Antigen retrieval was performed for 15 minutes in boiling pH 8 EDTA buffer (Abcam). The sections were allowed to cool to room temperature and then washed with PBS. The endogenous peroxidase was blocked with 3% hydrogen peroxide for 10 minutes. After washing with PBS, the sections were blocked with 20% goat serum diluted in PBS for 45 minutes. Sections were then incubated overnight at 4°C in a humidified chamber with primary antibodies diluted in 3% BSA. Primary antibodies used in this study are listed in Supplementary Table 2. The sections were washed with PBS and incubated with secondary antibodies for 1 hour at room temperature. Color visualization was performed with 3.3’-diaminobenzidine until the brown color fully developed. The sections were counterstained with hematoxylin, dehydrated, and coverslipped with permanent mounting media. The slides were scanned using the Aperio digital pathology slide scanner (Leica Biosystems). The images were analyzed using Aperio ImageScope software.

In situ hybridization (ISH) was performed using the RNAscope 2.5 HD Reagent Kit-BROWN (Advanced Cell Diagnostics) according to the manufacturer’s instructions. The following probes from Advanced Cell Diagnostics were used: *Lgr5* (cat. no. 312171) and *Lgr4* (cat. no. 318321).

BrdU staining was performed on formalin-fixed paraffin-embedded (FFPE) tissue sections using a monoclonal anti-BrdU antibody (MilloporeSigma) as described by the manufacturer. For Terminal deoxynucleotidyl transferase dUTP nick-end labelling (TUNEL) staining, the FFPE tissue sections were deparaffinized, treated with proteinase K and labeled using the In Site Cell Death Death Detection Kit POD (MilloporeSigma) according to the manufacturer’s instructions. To quantify the results of BrdU, TUNEL and RFP staining, thirty crypts/villi per mouse were scored for three mice in each group.

Myeloperoxidase (MPO) was used as the marker for neutrophils. Ten random-chosen 500 μm-length cecum sections were evaluated for each mouse. MPO^+^ cells within the band of lamina propria, immediately beneath and surrounding the crypts, were counted. Three mice in each group were analyzed. H&E-stained intestinal sections were evaluated for tumor stage by a board-certified GI pathologist (Dr. SF Kuan).

### Quantitative Reverse-transcription PCR analysis.

Total RNA was extracted from the mucosal layer of the mouse intestine or HT-29 cells using the RNeasy Mini Kit (Qiagen). The DNase-treated RNA was reverse-transcribed using SuperScript III reverse transcriptase (Invitrogen). The PCR reactions were performed on the CFX Connect Real-Time PCR Detection System (Bio-Rad Laboratories) using SsoAdvanced Universal SYBR Green Supermix (Bio-Rad Laboratories). The PCR thermal cycle conditions were as follows: denature at 95 °C for 30 s and 40 cycles for 95 °C, 10 s; 60 °C, 30 s. The specificity of the PCR products was determined by the melting curve analysis. *β-actin* was selected as an internal reference gene. The sequences of PCR primers are shown in Supplementary Table 3.

### Senescence-associated (SA) β-Galactosidase Staining.

After the mice were euthanized, the cecum was immediately removed and rinsed with ice-cold PBS. The tissues were frozen in dry ice after the excess liquid was carefully removed using filter paper. Then the tissues were embedded in OCT compound and cut into 10-μm sections. The assays were performed using the Senescence β-Galactosidase Staining Kit (Cell Signaling Technology) according to the manufacturer’s instructions. The sections were counterstained with hematoxylin before being dehydrated and coverslipped with mounting media.

### MSI Analysis.

The DNA was extracted from FFPE tissue sections using QIAamp DNA FFPE Tissue Kit (Qiagen). Cecal hyperplasia samples were from 6-week-old *FBC* mice. Cecal tumor samples were from 9–14.5-month-old *FBC* mice. Cecal tissue of 6-week-old *B* mice was used as control. According to a prior report ^62^, five microsatellite repeat markers, Bat24, Bat26, Bat30, Bat37 and Bat64, were used for MSI analysis. PCR amplification was carried out in a multiplex reaction using HSTaq polymerase (Takara Bio, Japan), with primer concentrations 0.5 μM. The thermal cycling conditions were as follows: initial denaturation at 95°C for 5 minutes; followed by 35 cycles of 95°C for 30 s, 60°C for 30 s, and 72°C for 30 s; then a final extension step at 68°C for 30 minutes. PCR fragments were analyzed by capillary electrophoresis, ABI3130XL (Life Technologies), and the GeneMapper ID3.2 program (Life Technologies). Tumor samples with greater or equal 40% MSI were classified as MSI-high (MSI-H), less than 40% as MSI-low (MSI-L), and samples without alterations were classified as MSS.

### RNA-seq and data analysis.

Total RNA was extracted from the cecal tissues of indicated mice using the RNeasy Mini Kit (Qiagen). After DNase I treatment and performing quality control (QC), 200 ng of high-quality total RNA was proceeded to library construction. Oligo(dT) magnetic beads were used to isolate mRNA. The mRNA was fragmented randomly by adding fragmentation buffer, then the cDNA was synthesized using mRNA template and random hexamers primer. Short fragments are purified and resolved with EB buffer for end repair and single nucleotide A (adenine) addition. After that, the short fragments were connected to sequencing adapters. The double-stranded cDNA library was completed through size selection and PCR enrichment. Agilent 2100 Bioanaylzer and ABI StepOnePlus Real-Time PCR System were used in the quantification and qualification of the sample library. Finally, the qualified RNA-seq libraries were sequenced using Illumina NovaSeq6000 in CD Genomics (Shirley, NY) after pooling according to its effective concentration and expected data volume. The FastQC tool was used to perform basic statistics on the quality of the raw reads. Sequencing adapters and low-quality data were removed by Cutadapt (version 1.17). The alignment tool Salmon (version 0.13.1) was employed to quantify transcript expression based on mm10 reference genome. Output files from Salmon were imported into R (V.4.2.0) and analyzed by DESeq2 package (V1.36.0) to identify differentially expressed genes. All genes were ranked by log2(fold change) and used to check the gene set enrichment by using clusterProfiler^[4]^ (V.4.4.1) in R. The following gene sets were used: MAPK signature ^20^; intestinal Wnt signature ^35^; cancer YAP/TAZ target gene signature ^36^; intestinal differentiation signature ^37^; intestinal stem cell signature ^38^; the Hallmark Inflammatory Response gene set (Broad Institute) ^34^; upregulated fetal spheroid markers ^41^; upregulated and downregulated genes in human SSA/P ^31^ (only genes in human SSA/Ps with fold increase>2 or fold decrease<−2 with FDR<0.05 were used).

### Whole exome sequencing.

DNA was extracted from the cecal tumor of 12-month-old *FBC* mice using DNeasy Blood & Tissue Kits (Qiagen). Sequencing libraries were generated using Agilent SureSelect mouse All Exon Kit (Agilent Technologies) following the manufacturer’s instructions and index codes were added to attribute sequences to each sample. DNA samples were sonicated using a hydrodynamic shearing system (Covaris) to generate 180–280bp fragments. The remaining DNA overhangs were converted into blunt ends by exonuclease/polymerase. After the adenylation of 3’ ends, DNA fragments were ligated with adapter oligonucleotides. The fragments with adapters on both ends were selectively enriched using PCR. Then the library was hybridized in the liquid phase with biotin-labeled probes, followed by the capture of the exons using streptomycin-coated magnetic beads. Captured libraries were enriched by PCR to add index tags to prepare for hybridization. The resulting products were then purified using the AMPure XP System (Beckman Coulter) and quantified using the Agilent High Sensitivity DNA Assay on the Agilent Bioanalyzer 2100 System. The qualified libraries were sequenced using Illumina NovaSeq6000 in CD Genomics (Shirley, NY) after pooling according to its effective concentration and expected data volume. For the alignment step, BWA is utilized to perform reference genome alignment with the reads contained in paired FASTQ files. For the first post-alignment processing step, Picard tools are utilized to identify and mark duplicate reads from BAM file. The variant calling was performed by using GATK HaplotypeCaller.

### Analysis of CRC patient data.

TCGA RNA-seq data and mutation data of all cancer types were collected from Xena database (https://xenabrowser.net/datapages/), i.e., TCGA Pan-Cancer (PANCAN), which includes 376 CRC tumor samples and 51 matched normal samples. Expression data for *FAK* and mutation data for *BRAF* were extracted for analysis. The difference between the two groups was evaluated using the Student *t*-test (two-tailed, pairwise).

## Data Availability

All data are available in the main text or supplementary materials.

## Significance statement

The “Goldilocks principle” in tumorigenesis suggests that optimum tumor development requires “just-right” levels of oncogenic signaling, with too much being as detrimental as too little. The *BRAF*^V8600E^ mutation is a driver mutation in colorectal tumorigenesis. We found no cecal tumor formation in *Vill-Cre*;*BRAF*^V600E^ mice, likely due to too much downstream ERK oncogenic signaling. Whether and how to tune BRAF^V600E^-induced ERK pathway activation *in vivo* has never been tested. This study found that FAK (focal adhesion kinase) loss downregulates BRAF^V600E^-induced ERK phosphorylation to increase intestinal stemness: the cecal tumor incidence increased from 0% in *Vill-Cre*;*BRAF*^V600E^ mice to 100% in *Vill-Cre*;*BRAF*^V600E/+^;*Fak*^fl/fl^ mice. This study provides the first *in vivo* evidence that mutant BRAF-induced ERK pathway activation can be tuned by FAK loss.
